# 遗传性嘧啶5′核苷酸酶缺乏症NT5C3A基因新发变异位点二例报告并文献复习

**DOI:** 10.3760/cma.j.issn.0253-2727.2021.08.012

**Published:** 2021-08

**Authors:** 园 李, 馨 赵, 向荣 胡, 建平 李, 佑祯 熊, 晓星 孙, 蕾 叶, 洋 杨, 洋 李, 文睿 杨, 广新 彭, 慧慧 樊, 康 周, 丽萍 井, 凤奎 张, 莉 张

**Affiliations:** 1 中国医学科学院血液病医院（中国医学科学院血液学研究所），实验血液学国家重点实验室，国家血液系统疾病临床医学研究中心，天津 300020 State Key Laboratory of Experimental Hematology, National Clinical Research Center for Blood Diseases, Institute of Hematology & Blood Diseases Hospital, Chinese Academy of Medical Sciences & Peking Union Medical College, Tianjin 300020, China; 2 亳州市人民医院血液内科，安徽亳州 236800 Bozhou People's Hospital, Bozhou 236800, China

遗传性嘧啶5′核苷酸酶（P5′N）缺乏症是一种罕见的红细胞酶异常相关的溶血性贫血，主因NT5C3A基因突变导致P5′N活动减低致病，以外周血涂片显著的嗜碱性点彩红细胞和红细胞内嘧啶核糖核苷酸蓄积为特征，呈常染色体隐性遗传模式。国际上首例临床诊断报道于1974年[1]，首例基因诊断报道于2001年[2]。目前全世界累计报道仅100余例，已报道的遗传性P5′N缺乏症NT5C3A基因突变位点达27个。我国虽有遗传性P5′N缺乏症的临床报道[3]–[5]，但尚无该病相关基因报道。我们于国内首次报道携带NT5C3A基因新发突变位点的遗传性P5′N缺乏症2例，并附文献复习。

## 病例资料

病例1，女，15岁，主因“巩膜黄染6年余”就诊。患者6年前发现巩膜黄染，未予重视。2年前腹部B超未见肝胆胰脾异常。1年前因“左肘内翻”矫形手术前查血常规发现HGB减低，WBC 3.91×10^9^/L、HGB 103 g/L、RBC 3.24×10^12^/L、PLT 285×10^9^/L、红细胞平均体积（MCV）100.1 fl、红细胞平均血红蛋白含量（MCH）31.8 pg，红细胞平均血红蛋白浓度（MCHC）318 g/L。血生化：总胆红素（TBIL）86.78 µmol/L，直接胆红素（DBIL）11.44 µmol/L，间接胆红素（IBIL）75.34 µmol/L，LDH 550 U/L。尿常规：尿胆原3+，潜血2+。未进一步诊治。8个月前腹部B超检出“胆囊多发结石”。1个月前因“胆石症、胆囊炎”行经内镜逆行性胰胆管造影（ERCP）取石术时疑诊“溶血性贫血”，为进一步诊治就诊于我院。个人史、月经史无特殊。家族中无类似病史，患者父母非血亲关系。入院查体：神志清，周身皮肤轻度黄染，全身浅表淋巴结无肿大。巩膜黄染，口唇无苍白。咽无充血，扁桃体未见肿大。胸骨无压痛，双肺呼吸音清，未闻及湿啰音，心音有力，律齐，各瓣膜区未闻及杂音。腹软，肝脏、脾脏肋缘下未触及。双下肢无水肿，神经系统查体未见异常。血常规：HGB 103 g/L，网织红细胞比值（RET）9.78％，网织红细胞绝对计数（ARC）0.315×10^12^/L，余正常。肝功能：TBIL 93.4 µmol/L，IBIL 83.1 µmol/L，LDH 556 U/L，余正常。P5′N活性1.05（参考值2.60～3.52）。其他溶血检查包括葡萄糖-6-磷酸脱氢酶（G6PD）活性、丙酮酸激酶活性、磷酸果糖激酶活性、红细胞渗透脆性试验、酸化甘油试验、伊红-5′-马来酰亚胺试验（EMA试验）、热不稳定试验、血红蛋白电泳、血红蛋白A2、血红蛋白F、异丙醇试验、变性珠蛋白小体试验、高铁血红蛋白还原试验、直接库姆试验、酸溶血试验等均阴性。未检出阵发性睡眠性血红蛋白尿（PNH）克隆。成熟红细胞寿命14 d。颅脑CT无异常。二代基因测序（图1）：（1）NT5C3A基因（转录本NM_016489）存在复合杂合突变：c.773T>C（p.I258T）和c.830A>G（p.D277G）,经Sanger测序技术家系验证发现c.773T>C（p.I258T）来于母亲，c.830A>G（p.D277G）来于父亲；（2）UGT1A1基因（NM_000463）存在纯合突变：c.211G>A（p.G71R）。未检出HBB、ALAS2等基因突变。诊断：1.遗传性P5′N缺乏症；2. Gilbert综合征。

病例2，女，54岁。主因“巩膜黄染、发现贫血10余年”就诊。外院检查血常规示HGB 90 g/L，肝肾功能：TBIL 110.9 µmol/L，IBIL 92.3 µmol/L，LDH 512 U/L，余正常。腹部B超未见脾肿大和胆结石。考虑“溶血性贫血”，曾应用泼尼松治疗3个月，贫血及黄疸无改善。为进一步诊治就诊于我院。既往史、个人史、月经史、婚育史无特殊。患者的父母为表亲，患者的奶奶和姥爷为同胞兄妹，均已故。查体：神志清，贫血貌，周身皮肤黄染，全身浅表淋巴结无肿大。巩膜黄染，口唇无苍白。咽无充血，扁桃体未见肿大。胸骨无压痛，双肺呼吸音清，未闻及湿啰音，心音有力，律齐，各瓣膜区未闻及杂音。腹软，肝脏、脾脏肋缘下未触及。双下肢无水肿，神经系统查体未见异常。血常规：HGB 88 g/L，RET 15.14％，ARC 0.354×10^12^/L，余正常。P5′N活性1.01（参考值2.60～3.52）。其他溶血检查包括G6PD活性、丙酮酸激酶活性、磷酸果糖激酶活性、红细胞渗透脆性试验、酸化甘油试验、EMA试验、热不稳定试验、血红蛋白电泳、血红蛋白A2、血红蛋白F、异丙醇试验、变性珠蛋白小体试验、高铁血红蛋白还原试验、直接库姆试验、酸溶血试验等均阴性。未检出阵发性睡眠性血红蛋白尿（PNH）克隆。经二代基因测序（图2）：（1）NT5C3A基因（转录本NM_016489）存在纯合突变：c.252+1G>A，患者父母已故，无法完成家系验证，患者之子NT5C3A基因携带单等位基因杂合突变c.252+1G>A；（2）UGT1A1基因（NM_000463）存在杂合突变c.211G>A（p.G71R）。未检出HBB、ALAS2等基因突变。诊断：遗传性P5′N缺乏症。

**图1:**
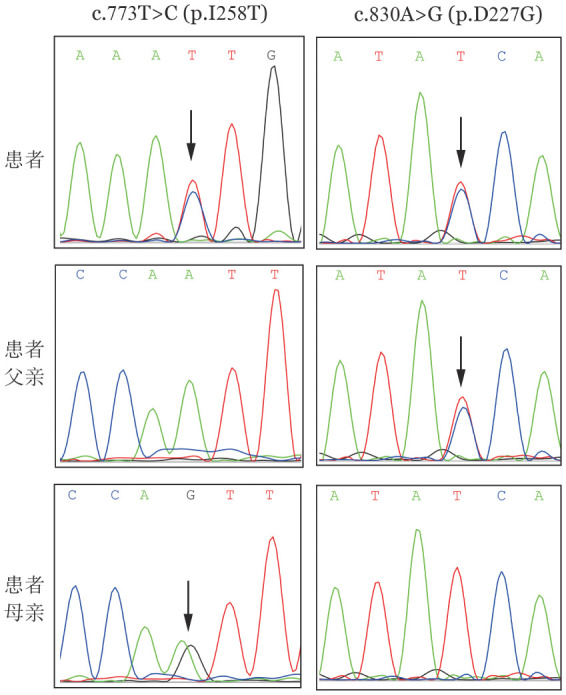
病例1的NT5C3A基因突变测序图（箭头所示为突变位点）

**图2:**
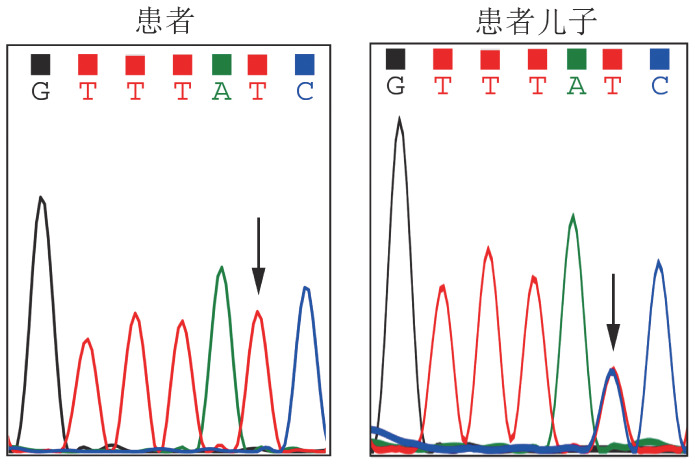
病例2的NT5C3A基因突变测序图（箭头所示为突变位点）

## 讨论

在先天性红细胞酶病所致的溶血性贫血中，遗传性P5′N缺乏症是第三常见的疾病，排在G6PD缺乏症和丙酮酸激酶缺乏症之后[6]。由于该病罕见，确切的发病率不详，诊断亦常延误，中位诊断年龄为15岁（3月龄～64岁）[7]。

遗传性P5′N缺乏症为常染色体隐性遗传性疾病，其致病基因NT5C3A位于染色体7p14，包含11个外显子[2]。NT5C3A基因产物为P5′N1型（P5′N-1），也称为尿嘧啶单核苷酸水解酶1型（UMPH-1），该酶主要表达于成熟阶段红细胞，将嘧啶单核苷酸水解为嘧啶核苷。突变导致活性P5′N-1缺失[7]–[8]，从而出现红细胞内嘧啶核苷酸堆积，发生溶血。

该病主要临床表现为轻、中度贫血，重度贫血约占12％[7]。黄疸和脾肿大也较常见（分别为46/61、49/62），约1/3的患者出现胆石症和肝肿大，约1/6的患者出现过新生儿黄疸。患者的中位HGB 95（28～152）g/L，未切脾者的RET约8％，IBIL 47 µmol/L。外周血涂片可见2％～12％的嗜碱性点彩红细胞。外周血红细胞P5′N-1活性（*A*_260_/*A*_280_）常减低至1％～64％。

该病的临床和血液学特征显著但不特异，临床诊断主要取决于红细胞内高浓度的嘧啶核苷酸和P5′N-1活性下降[7]。检出NT5C3A基因纯合突变或复合杂合突变，有助于精准诊断该病[9]–[10]。且须排除其他可能引起P5′N-1活性下降的疾患，例如β型地中海贫血、血红蛋白病、铁粒幼细胞贫血、铅中毒等。

本文中的2例患者均为明确的溶血性贫血，长期中度贫血，P5′N-1活性下降，分别检出了NT5C3A基因纯合突变或复合杂合突变。依据患者的病史、家族史、血液学检查、P5′N-1活性测定、NT5C3A基因检测情况，可明确诊断为遗传性P5′N缺乏症。

通过查询ClinVar数据库和HGMD数据库，本文中c.773T>C（p.I258T）为已知致病性突变[11]，其他两个突变位点c.830A>G（p.D277G）和c.252 + 1G>A均为新发突变。c.830A>G（p.D277G）位于NT5C3A基因第10外显子，突变导致277位的天冬氨酸（酸性氨基酸）替代为甘氨酸（非极性、疏水性氨基酸），Swiss-model模拟P5′N-1蛋白三级结构可见侧链消失（图3），推测突变位点可能影响了P5′N-1的酶催化效能或热稳定性，继而降低酶的活性。而c.252+1G>A位于第5外显子的高度保守区，邻近于已知的突变位点c.251dupA[12]–[13]。c.251-252insA造成第83位密码子插入了1个碱基，P5′N-1的酶活性降至正常人的27.5％。推测本文中的剪接突变c.252+1G>A可能导致NT5C3A基因部分功能丧失，破坏酶的活性。依据ClinVar数据库，目前已报道的突变位点共27个，分别为错义突变9个，缺失突变6个，剪接突变5个，插入突变4个，无义突变3个。未检索到我国关于该病致病基因的报道。

**图3:**
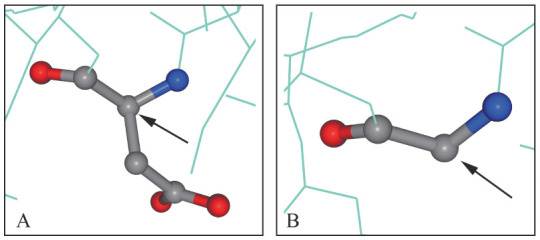
NT5C3A基因的野生型和突变型蛋白结构模拟图 A：NT5C3A基因野生型c.830A（p.D277）蛋白结构模拟，可见侧链结构完整（箭头处）；B：NT5C3A基因突变型c.830A>G（p.D277G）的蛋白结构模拟，可见侧链消失（箭头处）

这两例患者都携带UGT1A1基因突变c.211G>A（p.G71R）[14]。例1为纯合子，合并Gilbert综合征，例2为杂合子，类似情况曾见于Santos等的报道[15]。据我们所知，这可能也是国内首次报道遗传性P5′N缺乏症合并Gilbert综合征。明确合并的UGT1A1基因突变的情况，有益于更准确地分析NT5C3A基因型-临床表型的关系。Gilbert综合征[16]为一种较常见的遗传性胆红素代谢性疾病，呈常染色体隐性遗传模式，致病基因UGT1A1基因编码葡萄糖醛酸转移酶，该酶可使IBIL转变成DBIL。当UGT1A1基因突变，其编码的酶功能受损，DBIL生成减少，IBIL水平增高。临床表现为慢性轻、中度的IBIL增高，无临床症状，也无溶血和肝损伤的证据。与溶血性贫血合并存在时，血清IBIL水平将进一步增高。由于与溶血性贫血的临床表型重叠，当与溶血性贫血合并存在时，Gilbert综合征极易被忽略和漏诊。在诊断溶血性贫血时，如溶血严重程度与血清胆红素水平不相称，且无明确的肝损伤时，需注意合并Gilbert综合征等遗传性胆红素代谢性疾病。通过二代基因测序技术检测覆盖相关致病基因突变，可明确诊断[17]。

本文于国内首次报道了该病的基因突变，并且报道了2个新发突变位点，也是我国关于遗传性P5′N缺乏症合并Gilbert综合征的首次报道。新发位点丰富了NT5C3A基因突变的数据，对于该病的基础和临床研究、精准诊断、甚至靶向治疗都将起到积极作用。
